# A Case of Rheumatic Chorea

**Published:** 1856-10

**Authors:** Henry Hartshorne


### *A Case of Rheumatic Chorea.*

By Henry Hartshorne, M. D.

Allusion having been made, in the interesting article of Dr. Gibbs upon Chorea, in the September number of the Examiner, to the *rheumatic* affinities of that disease, I present the following case, as affording, in a more than usually clear manner, an illustration of the occasional connection of the two disorders.

Ann Cook, aged 13, and previously healthy, was, after some household exposure to wet and cold, attacked, about the 8th of February, 1856, with acute articular rheumatism. It affected chiefly the ankles, which were much swollen, hot and painful. I was not called for several days after the commencement of the attack; but found her, then, suffering very much with the inflamed joints, and with considerable fever. A saline purge was advised, after which she took the vin. colchic. rad. in ordinary doses, with magnesia. No application was made to the feet, except laudanum. On the 14th, the articular affection entirely, and almost suddenly, subsided; with an increased violence of the pulse; and especially of the impulse of the heart, a flushed face, orthopnœa, and distressing pain in the cardiac region. A bellows murmur was heard, with the second sound of the heart: whether this had been present at any previous time, no examination had determined. Four or five ounces of blood were taken by cups from between the shoulders, followed by a blister over the sternum; and tinc. digital. was added to the colchicum mixture, which she continued to take. The violence of her symptoms was thus materially relieved; and, by the 22nd, I pronounced her well of the carditic attack, without any return of the articular rheumatism. A very slight bellows murmur only remained, with no functional derangement of the heart.

On the 1st of March, I was sent for, to treat her for *chorea*, which had gradually commenced almost from the time of cessation of the cardiac symptoms above alluded to, and had now assumed a violent form. The irregularity and spasmodic frequency of movement affected the muscles of the whole body, including the tongue, face and neck,—so that she could not speak intelligibly; nor could she walk across the room without help. All of these movements subsided (as usual), entirely, during sleep.

She was, at this time, pale, rather thin, feeble, and without appetite. The treatment pursued was the usual routine of cimicifuga and mineral tonics, commencing the latter series with iron, and ending with sulphate of zinc. Salt baths, frequent dry rubbing, and friction on the spine with croton oil, were also employed.

The improvement, however, was very gradual, not approaching convalescence until about the close of April. She was then made worse by a severe fright; but continued, nevertheless, after a short interval, to improve, and was well by the early part of June.

The only particular interest in this case was the close and apparently, metastatic, succession of the three forms of disease, viz: the articular rheumatism, rheumatic carditis, and chorea. Each of these gave way just before another appeared. Except in the more strongly marked character of the cardiac inflammation, it resembled somewhat Case LXXII related by Dr. Todd (Nervous System, Phila. Ed., p. 281). While it illustrates, as we have remarked, the statements made by many clinical observers, as to the frequent association of chorea with the rheumatic diathesis, it does not at all tend to confirm the hypothesis of Simon (quoted in the paper of Dr. Gibbs referred to, Exam., Sept. 1856) in regard to the order in which the "irritation" of chorea and the "explosion" of rheumatism, are likely to occur.

Perhaps the best hypothetical mode of stating the view which the case I have narrated suggests, is—that the mobile cause (whatever its nature) to the effects of which we give the name of rheumatism, when shown in the articular, cardiac, or febrile symptoms, had, upon leaving its seat in the fibrous tissues of the heart, attacked those of the nerve-centres, sensorial or supraspinal; in the functional disturbance of which the choreic disorder consisted. It would seem thus to be analogous to the cases of *rheumatic paralysis,* so often observed.

It may not be out of place to remind the reader, that in the observations of Drs. Hughes and Burton Brown (Guy's Hospital Reports, 1846 and 1855), upon 309 cases of chorea in Guy's Hospital, they referred to rheumatism as the actual *exciting*

*cause*, in 13 cases; while in 58 cases where inquiry was made in regard to it, 30 had suffered from rheumatic attacks; and of 104, only 15 were free both from cardiac murmurs and from the influence of previous attacks of rheumatism.

These facts show the importance of the rheumatic diathesis in predisposing to chorea; while they do not of course controvert the generally received opinion, that disorders of nutrition, and morbid impressions upon the nervous system, account for it in a still greater number of cases.

